# Case report: pericardial effusion with constrictive physiology in a patient with wet beriberi

**DOI:** 10.1186/s12937-016-0156-y

**Published:** 2016-04-08

**Authors:** Minako Yamamura, Hisayoshi Murai, Shuichi Kaneko, Soichiro Usui, Hiroshi Furusho, Masayuki Takamura

**Affiliations:** Disease Control and Homeostasis, Graduate School of Medical Science, Kanazawa, University, 13-1 Takara-machi, Kanazawa, 920-8641 Japan

**Keywords:** Beriberi, Heart failure, Pericarditis, Constrictive physiology

## Abstract

Wet beriberi-induced pericardial effusion has rarely been previously described. Little is known about the effect of beriberi-induced pericardial effusion on hemodynamics. Here we present a case of wet beriberi with pericardial effusion that exhibited constrictive physiology, which was dramatically improved after treatment. A 61-year-old male patient was admitted to our hospital for progressive leg edema, dyspnea on exertion, and lower-extremity muscle weakness. Echocardiography showed a hyperkinetic left ventricle and a moderate amount of pericardial effusion. Hemodynamic measurements, including simultaneous measurement of left and right ventricular pressures, revealed high output heart failure and constrictive physiology. Blood test showed lactic acidosis, and low level of serum thiamine levels; consistent with a diagnosis of wet beriberi. After thiamine replacement therapy, the patient’s hemodynamic state rapidly improved. Additionally, pericardial effusion decreased and constrictive physiology was successfully resolved. No other possible causes of pericardial effusion could be identified, with the exception of thiamine deficiency. This case illustrates the importance of considering wet beriberi as a possible cause of pericardial effusion with constrictive physiology.

## Background

Wet beriberi is one of the clinical syndromes associated with thiamine (Vitamin B1) deficiency, and is typically characterized by high output heart failure with low peripheral vascular resistance [1]. Its most fulminant form is shoshin beriberi, which is characterized by cardiovascular collapse and these patients may die within hours if left untreated [2, 3]. Unfortunately, wet beriberi often goes unrecognized, a consequence of its rarity and the non-specificity of symptoms [3]. Clinical presentation may be somewhat varied and pericardial involvement is rarely manifested. Little is known regarding the effect of wet beriberi-related pericardial effusion on hemodynamic parameters. Here we report the case of a wet beriberi patient accompanied by pericardial effusion with constrictive physiology; equalization of end diastolic pressure in right and left ventricular with dip and plateau pattern and increased ventricular interdependence [4], and whose condition rapidly improved after the replacement of thiamine.

## Case presentation

**Fig. 1:**
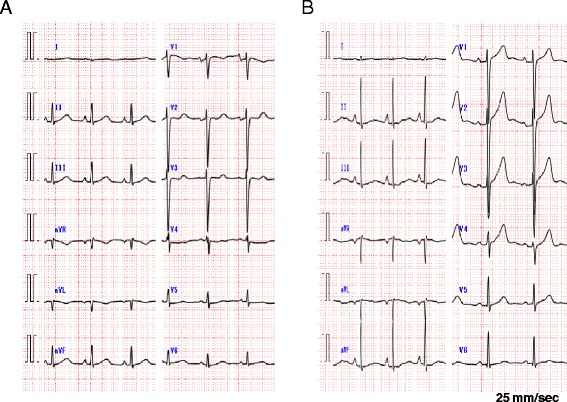
Electrocardiogram on admission showed slightly low voltage, poor progression R wave and ST segment depression in leads V4-5 (**a**). With the exception of the poor progression R wave, all changes were no longer observed by day 27 (**b**)

**Fig. 2:**
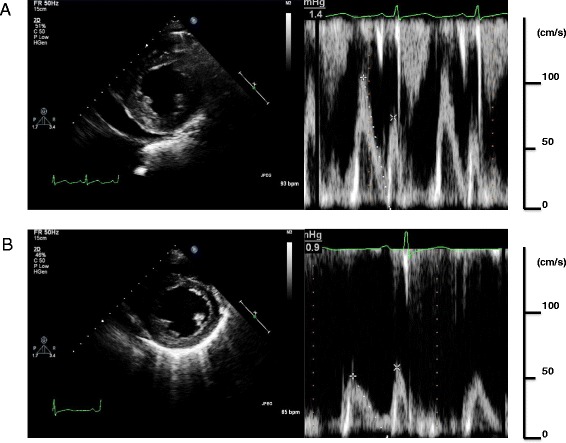
A transthoracic echocardiogram showed a moderate amount of pericardial effusion. A pseudonormal pattern of transmitral flow was observed on administration of thiamine (**a**). Two days after thiamine administration, pericardial effusion decreased and the mitral Doppler findings showed that constrictive physiology was resolved after thiamine replacement therapy (**b**)

A 61-year-old male patient was admitted to our hospital with progressive external dyspnea, leg edema, and muscle weakness. The patient was in good health up to a point two months prior to admission. The patient had a history of stomach ulcers and drank alcohol socially. He ate an unbalanced diet for many years. His BMI was 20.2 kg/m^2^ and blood pressure was 97/45 mmHg. Paradoxical pulsation was not observed. The patient’s heart rate was 92 bpm and oxygen saturation was 98 % at room air. A physical examination revealed jugular venous distention, although heart and breath sounds were unremarkable. Bilateral pitting edema was observed throughout the legs and muscle tenderness in the lower extremities was noted. A chest X-ray showed cardiomegaly, pulmonary vascular congestion, and pleural effusion. An electrocardiogram revealed ST segment depression in leads V4-5 (Fig. 1a). Echocardiography revealed a hyperkinetic left ventricular (LV) ejection fraction of 85 % without regional wall motion abnormalities and normal LV size (end-diastolic dimensions: 53 mm). A moderate amount of pericardial effusion was noted and mitral Doppler measurements showed the peak E wave velocity of 112 cm/s, the E/A ratio of 1.6, and E wave deceleration time of 151 ms; consistent with a pseudonormal pattern. No significant respiratory E wave variation (17 %) was observed (Fig. 2a). Tricuspid regurgitation was observed with a maximal velocity (TR V max) of up to 3.4 m/s. The inferior vena cava was markedly dilated to 23 mm and respiratory variation was diminished. Arterial blood gas analysis showed severe metabolic acidosis with an elevated lactate level of 56 mg/dL, while pH was preserved by a compensatory hyperventilation response. B-type natriuretic peptide (BNP) levels were elevated to 322.8 pg/mL. No evidence of any viral infection or mycobacterium tuberculosis, both possible cause pericarditis, was found. Thyroid function was also normal. In addition, no malignancies were observed and all immunological profiles were negative.

**Fig. 3:**
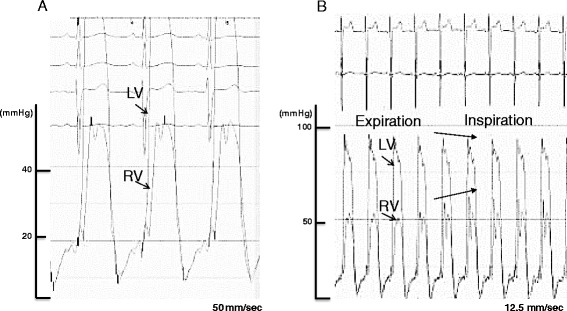
Simultaneous right and left ventricular measurements showed elevated and approximately equal end-diastolic pressures with dip and plateau pattern (**a**). Paradoxical pulsation was not observer during respiratory change, but respiratory ventricular discordance was seen; Arrows shows increased RV pressure occurred concomitantly with decreased LV pressure decreased during inspiratory phase (**b**)

Intravenous furosemide was administered to the patient, however, his condition failed to improve. Hemodynamic evaluation using a Swan-Ganz catheter revealed high output heart failure with low peripheral vascular resistance and constrictive physiology, cardiac index of 5.35 l/min/m^2^, pulmonary capillary wedge pressure of 18 mmHg, and systematic vascular resistance of 400 dyne・sec・cm^-5^. Right ventricular (RV) and LV end diastolic pressures were approximately equal (20 and 19 mmHg, respectively) and exhibited a dip and plateau pattern (Fig. 3; Table 1). Coronary angiography was normal.

**Table 1:** Hemodynamic data from cardiac catheterization

Variable	Value	
	day4	day27
Pressures (mmHg)		
Right atrium (a/v/mean)	20/19/15	6/5/4
Right ventricle	53/20	23/6
Pulmonary artery	51/20	25/10
Pulmonary capillary wedge	18	10
Left ventricle	85/19	134/13
Aorta	86/49	146/76
Cardiac output (l/min)	10.0	6.47
Cardiac index (l/min/m^2^)	5.35	3.93
Systematic vascular resistance (dyne・sec・cm^-5^)	400	1236
Pulmonary vascular resistance (dyne・sec・cm^-5^)	128	87

Given the deficiency of thiamine (measured concentration: 13 ng/mL; normal range: 24-66 ng/mL) and the presence of lactic acidosis, we suspected wet beriberi in this case. Intravenous thiamine was administered (100 mg/day). Approximately seven hours after initial administration, the patient’s urine output rapidly increased and his hemodynamic status improved dramatically. Two days after thiamine administration, an echocardiograph showed normal LV contraction (EF: 66 %), decreased pericardial effusion, and an improved pseudonormal pattern by mitral Doppler examination. The E/A ratio decreased to 0.8 and E wave deceleration time was extended to 204 ms (Fig. 2b). Lactic acidosis resolved 47 h after first dose and heart failure symptoms ceased after 2 days of thiamine replacement therapy. Twenty days after thiamine administration the hemodynamic profile was as follows: cardiac index 3.93 l/min/m^2^, pulmonary capillary wedge pressure 10 mmHg, and systematic vascular resistance 1236 dyne・sec・cm^-5^. Simultaneous RV and LV pressure tracings showed decreased end diastolic pressures, while respiratory ventricular discordance was negative. Electrocardiogram showed all changes were no longer observed by day 27 with the exception of the poor progression R wave (Fig. 1b). This rapid recovery post-administration of thiamine provided strong support for a diagnosis of wet beriberi. Cardiac magnetic resonance imaging was done in order to investigate the cause of the constrictive physiology. However, late gadolinium enhancement of the myo- and pericardia, increased pericardial thickening, and calcification were not observed. In addition, no pathological findings related to the pericardium were identified. As the pericardial effusion rapidly decreased and the constrictive physiology resolved after thiamine administration, we concluded that wet beriberi was responsible for the pericardial involvement. This could further have a restraining effect on both ventricles, thus accounting for the constrictive physiology observed. On follow-up, no recurrence of pericardial effusion was found. Echocardiography was performed 47 days after initiation of intravenous thiamine and this confirmed successful resolution of the constrictive physiology.

## Discussion

Wet beriberi is one of the clinical syndromes associated with thiamine deficiency and is hemodynamically characterized by high output heart failure with low peripheral vascular resistance [1]. Thiamine is a water-soluble vitamin that serves as a cofactor for two key enzymes in the tricarboxylic acid cycle: pyruvate dehydrogenase and alpha-ketoglutarate dehydrogenase. Thus, a deficiency of thiamine leads to decreased enzyme activity and consequent anaerobic metabolism and lactic acidosis, a condition that can be reversed by thiamine replacement therapy [2]. Although the precise mechanism remains unknown, impaired myocardial energy metabolism does result in myocardial damage, while a thiamine deficiency leads to decreased systemic vascular resistance. This myocardial dysfunction and increased preload can result in congestive heart failure [5]. Increased pulmonary arterial blood flow, elevated pulmonary capillary wedge pressure reflecting elevated LV end-diastolic pressure, and elevated pulmonary vascular resistance can all cause pulmonary hypertension [6]. A striking and complicating clinical feature in this case was the constrictive physiology observed in addition to high cardiac output with low peripheral vascular resistance.

Beriberi occurs mainly in patients with inadequate nutrition and chronic excessive alcohol intake [7, 8]. Currently, diagnosis is dependent on the following factors: a characteristic history of dietary inadequacy in combination with excessive alcohol intake, exclusion of other etiologic types of heart disease, additional signs of thiamine deficiency, and therapeutic response to thiamine administration [9]. Administration of thiamine is recommended for both the diagnosis and treatment of beriberi. In our case, a decrease in serum concentrations of thiamine, lactate acidosis, and the dramatic improvement of clinical features after thiamine replacement all lead to a final diagnosis of wet beriberi. However, the accurate etiology for beriberi remained unclear. His unbalanced nutrition and poor appetite with gastric ulcers might have induced the deficiency of thiamine leading to wet beriberi.

The clinical presentation of beriberi is often variable. Constrictive physiology, as observed in our patient, appears to be an extremely rare clinical manifestation of this disease. Thus, we initially investigated all possible etiologies of this finding before finally considering wet beriberi. However, after careful consideration of additional possible causes and the clinical course observed (resolution of pericardial effusion and constrictive physiology after thiamine administration), we ultimately concluded that pericardial effusion was a result of increased hydrostatic pressure brought about by wet beriberi. This in turn resulted in the restraining effect on both ventricles, mimicking the hemodynamic state of constrictive pericarditis. Araki et al. previously reported a case of beriberi heart disease with exudative pericardial effusion [10]. They speculated that pericardial effusion was associated with beriberi based on the clinical course of their patient, although the precise mechanism was unknown. In our case, as no pathological findings related to pericarditis were identified, it is reasonable to speculate that a non-inflammatory transudative pericardial effusion was caused by wet beriberi. In this case, the severe leg edema could be partly attributable to RV heart failure with constrictive physiology.

## Conclusions

Here we presented the case of a patient with wet beriberi accompanied by constrictive physiology, whose condition improved dramatically with timely treatment. Clinical presentation may be somewhat varied and pericardial involvement is rarely manifested. To the best of our knowledge, this is the first report of a wet beriberi case presenting with constrictive physiology. The case presented here should serve to remind clinicians of the importance of considering wet beriberi as a possible cause of pericardial effusion with constrictive physiology.

## Consent

Written informed consent was obtained from the patient for publication of this Case Report and any accompanying images. A copy of the written consent is available for review by the Editor-in-Chief of this journal.

## Abbreviations

LV: Left ventricular
BNP: B-type natriuretic peptide
RV: Right ventricular
